# The Synergistic Effect of Biosynthesized Silver Nanoparticles and Phytocompound as a Novel Approach to the Elimination of Pathogens

**DOI:** 10.3390/molecules28237921

**Published:** 2023-12-04

**Authors:** Natalia Wrońska, Sara Płaczkowska, Katarzyna Niedziałkowska, Katarzyna Lisowska

**Affiliations:** Department of Industrial Microbiology and Biotechnology, Faculty of Biology and Environmental Protection, University of Lodz, 12/16 Banacha Street, 90-236 Lodz, Poland; sara.placzkowska@edu.uni.lodz.pl (S.P.); katarzyna.niedzialkowska@biol.uni.lodz.pl (K.N.); katarzyna.lisowska@biol.uni.lodz.pl (K.L.)

**Keywords:** silver nanoparticles biosynthesis, *Trametes versicolor*, antimicrobial activity, synergistic effect, phytocompound

## Abstract

Due to the wide applications of silver nanoparticles (AgNPs), research on their ecological synthesis has been extensive in recent years. In our study, biogenic silver nanoparticles were synthesized extracellularly using the white rot fungus *Trametes versicolor* via two cultivation methods: static and shaking. The cell filtrate of the fungus was used as a reducing agent in the process of nanoparticle synthesis. Characterization of the obtained nanoparticles was carried out using UV–VIS spectroscopy and scanning electron microscopy. The biosynthesized nanoparticles have antimicrobial potential against pathogenic bacteria, particularly in Gram-negative strains. The bactericidal effect was obtained for *E*. *coli* at a concentration of 7 µg/mL. The use of higher concentrations of compounds was necessary for Gram-positive bacteria. Taking into account the problem of the risk of cytotoxicity of AgNPs, combined therapy using a phytochemical was used for the first time, which was aimed at reducing the doses of nanoparticles. The most representative synergistic effect was observed in the treatment of 5 µg/mL silver nanoparticles in combination with 15 µg/mL ursolic acid against *E. coli* and *P. aeruginosa* with a bactericidal effect. Moreover, the coadministration of nanoparticles considerably reduced the growth of both *Staphylococcus* strains, with a bactericidal effect against *S. aureus.* The viability test confirmed the strong synergistic effect of both tested compounds. Silver nanoparticles synthesized using the *T. versicolor* showed excellent antibacterial potential, which opens perspectives for future investigations concerning the use of the nanoparticles as antimicrobials in the areas of health.

## 1. Introduction

Nanotechnology has revolutionized various scientific fields, including medicine, electronics, and environmental science. One of the most important advancements in this field is the synthesis of nanoparticles. Silver nanoparticles (AgNPs) have attracted significant interest due to their unique physical, chemical, and biological properties. AgNPs exhibit exceptional catalytic, electrical, and antimicrobial activities, making them suitable for a wide range of applications. They have been extensively used as antibacterial agents in the pharmaceutical industry, industrial household and healthcare-related products, cosmetics, medical device coatings, the food industry, diagnostics, and drug delivery [1,2]. Some studies also indicate the successful use of AgNPs as plasmonic sensors for water pollutants such as heavy metals and organic compounds and as suitable photocatalysts to promote the oxidative degradation of dyes and pesticides, which enables their environmental applications [3,4,5,6,7]. 

Three main methods of nanoparticle synthesis are mentioned in the literature: chemical, physical, and biological [8]. Conventional chemical and physical methods seem to be very expensive and hazardous [9,10]. Climate change and global warming are stimulating the scientific community to introduce environmentally friendly synthesis methods. In the context of green chemistry, biological methods using microbes have an advantage over the other two. Among the various methods available, the use of biological agents, such as fungi, has gained considerable attention. Fungi, being eukaryotic microorganisms, possess unique capabilities to interact with metal ions and reduce them to form metallic nanoparticles. The use of fungi for synthesizing AgNPs offers several advantages, including their eco-friendliness, cost-effectiveness, and the ability to produce nanoparticles with a narrow size distribution. Moreover, this process coats nanoparticles with biomolecules derived from the fungus, which may improve stability and enhance biological activity [11,12]. 

Various fungus species may be used in biogenic synthesis, enabling the production of AgNPs with different properties. Certain fungi species, such as *Trichoderma*, *Aspergillus*, *Penicillium*, and *Fusarium*, have been studied for their ability to produce AgNPs [13]. The fungus strain is selected based on its metal tolerance, growth rate, and ability to produce nanoparticles. The economics of the process are also important; therefore, microorganisms with low cultivation requirements are sought. The synthesis efficiency can be optimized by adjusting parameters such as pH, temperature, silver precursor concentration, biomass amount, and cultivation time. 

The synthesis of nanoparticles using fungi can occur in two ways: in vivo and in vitro. The first method takes place inside the fungal cells and is a kind of defense mechanism of the fungi against metal ions that are reduced to nanoparticles. The disadvantage of the method is the need to extract nanoparticles. In vitro production of nanoparticles includes three methods. The first method involves the use of fungal supernatants containing enzymes secreted into the growth medium. In the next method, silver nanoparticles are synthesized using post-culture extracts of fungi. The third way includes using the aqueous solution obtained after the incubation of a fungal culture in pure water [14]. Given that fungi are extremely efficient secretors of extracellular enzymes, it is thus possible to easily obtain large-scale production of nanoparticles [15].

Silver nanoparticles (AgNPs) have gained significant attention due to their exceptional antimicrobial properties. AgNPs synthesized using fungi have demonstrated broad-spectrum antibacterial activity against both Gram-positive and Gram-negative bacteria. They have been effective against pathogenic bacteria such as *Escherichia coli*, *Staphylococcus aureus*, *Pseudomonas aeruginosa*, and *Salmonella* spp. The antibacterial activity of AgNPs is not limited to specific strains and is generally effective against a wide range of bacterial species, including antibiotic-resistant strains. The antibacterial properties of AgNPs are attributed to their unique physicochemical properties and interaction with bacterial cells. The small size and large surface area of AgNPs facilitate their efficient penetration into bacterial cells. Once inside, AgNPs interact with various cellular components, disrupting essential processes and leading to bacterial cell death. The main mechanisms of antibacterial action include membrane damage, reactive oxygen species (ROS) generation, protein denaturation, and DNA damage [16,17,18].

It has been reported that properties such as shape, size, surface charge, and diffusion state can affect the antimicrobial activity of silver nanoparticles (AgNPs) [19,20,21]. It is suggested that smaller nanoparticles (<10 nm) can penetrate bacterial cells more easily. One of the described mechanisms of antibacterial action of AgNPs involves their adhesion to the cell membrane, disturbing its permeability and respiration function of bacterial cells [16,22,23]. Nanoparticles can also penetrate inside the bacteria cells [17]. AgNPs interact with sulfur-containing proteins present in bacterial membranes and with phosphorus-containing compounds (e.g., DNA) [24]. When nanoparticles enter the bacterial cells and attack the respiratory chain, cell division finally leads to cell death. Silver ions released in bacterial cells increase the bactericidal effect of nanocompounds [17,18,25].

The potent antibacterial properties of silver nanoparticles synthesized using fungi hold promise for numerous applications in biomedical and environmental settings. In the medical field, they can be utilized in wound dressings, medical implants, and coatings to prevent bacterial infections. AgNPs can also be incorporated into dental materials, such as composites and adhesives, to inhibit the growth of oral bacteria.

The current study was designed to evaluate the antimicrobial activity of nanoparticles and their synergistic effect with the selected triterpenoid–ursolic acid. The combination of silver nanoparticles and ursolic acid can reduce their cytotoxicity and improve their therapeutic effectiveness at low doses. Triterpenoids are the most abundant group of terpenoids found in dicotyledonous plants. These compounds are involved in the adaptation of plants for survival and function as specific chemical weapons against competitive plants, pathogens, or herbivores. Some studies have described the anti-inflammatory [26], antioxidant [27], antibacterial [28,29,30], anticancer [31,32], and antiparasitic [33] potential of triterpenes. The representative pentacyclic triterpenoid is ursolic acid (UA). Its activities can also enhance bacterial susceptibility to other compounds [34]. 

In this paper, the antimicrobial properties of nanoparticles produced in an eco-friendly manner using the white rot fungus *Trametes versicolor* are described for the first time. The main idea of the study was to synthesize AgNPs without using toxic chemicals such as capping or stabilizing agents.

## 2. Results

### 2.1. Eco-Friendly Silver Nanoparticles Synthesis

**Figure 1:**
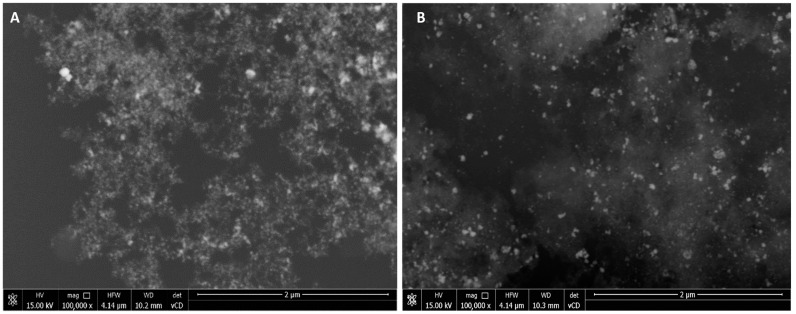
SEM images of AgNPs: (**A**) AgNPs 1—from static culture and (**B**) AgNPs 2—from shaking culture.

Silver nanoparticle (AgNP) synthesis was carried out using the *Trametes versicolor.* Two cultivation methods were carried out: static and shaking. Two suspensions of newly synthesized silver nanoparticles were obtained: AgNPs 1—from static culture and AgNPs 2—from shaking culture. The UV–VIS spectrum of the post-culture liquid supplemented with AgNO_3_ demonstrated a plasmon band at 425 nm, which is the characteristic band of metal nanoparticles. A sample from each variant was dried and applied to a silicon wafer for analysis using a scanning electron microscope (SEM). The results of the analysis are shown in Figure 1.

Based on the SEM image, it was found that silver nanoparticles (AgNPs) produced using the filamentous fungus *Trametes versicolor* have heterogeneous shapes and sizes. Moreover, the images obtained for AgNPs 1 and AgNPs 2 suggest that the nanoparticles are covered with an extracellular substance produced by the tested microorganism. Therefore, detailed analysis of the shape of nanoparticles was difficult. It has been proven that during the synthesis process, nanoparticles are coated with biomolecules derived from the fungus, which can improve stability and confer biological activity [11].

The progress of the ecological approach to the synthesis of silver nanoparticles is a major step in green nanotechnology, enabling their applications in nanomedicine. The synthesis of nanomaterials in a safe and environmentally friendly way is the main goal of green nanotechnology. Biosynthesis of nanomaterials using microorganisms or plants has been recognized as a sustainable and cheaper alternative to chemical and physical methods [14,35]. The ability of fungi from the genera *Penicillium*, *Aspergillus*, *Trichoderma,* and *Fusarium* to synthesize silver nanoparticles has been described [36,37,38]. Filamentous fungi can synthesize metal nanoparticles of various shapes, sizes, and properties [14]. White rot fungi *Trametes versicolor*, due to their strong enzymatic system, are suggested to be microorganisms capable of forming metallic nanoparticles [39]. However, there are little data in the literature. Therefore, in this work, silver nanoparticles were obtained using *T. versicolor* post-culture liquid. Visualization of the obtained compounds, using scanning electron microscopy, showed that the nanoparticles are coated with a substance secreted by the fungus. Due to the strong foaming of the *T. versicolor* filtrate, we assume that these were surfactants. UV–VIS analyses of AgNPs 1 and AgNPs 2 revealed a peak with maximum absorption at 425 nm, which is the result of surface plasmon resonance typical for silver nanoparticles. A similar result was observed in the UV–VIS spectrum of AgNPs produced using *Rhizopus stolonifera* [40]. The peak at 440 nm was observed in the UV–VIS spectrum of AgNPs synthesized using *Aspergillus tubingensis* and *Bionectria ochroleuca* [41].

### 2.2. Antimicrobial Activity of Silver Nanoparticles

**Figure 2:**
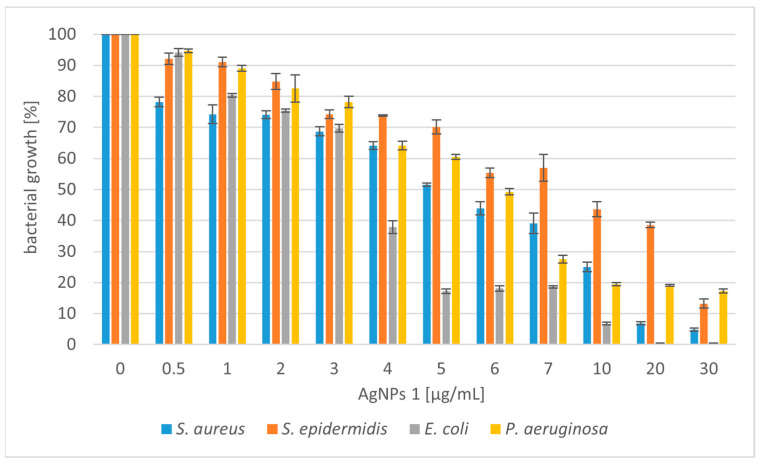
Reduction in bacterial growth after AgNPs 1 treatment and incubation for 24 h.

**Figure 3:**
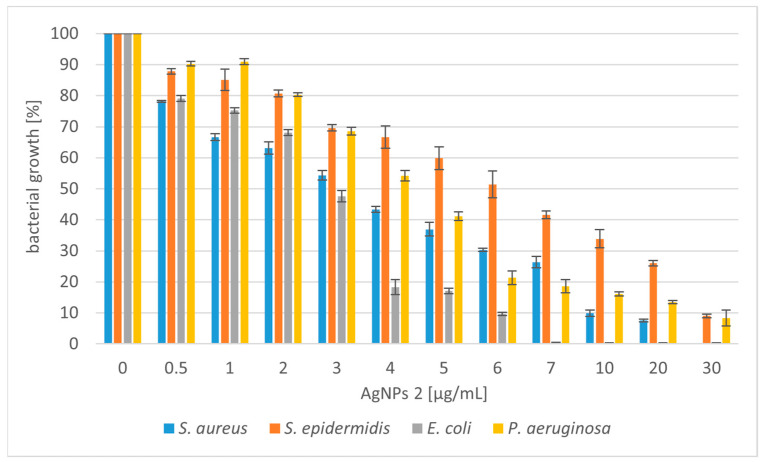
Reduction in bacterial growth after AgNPs 2 treatment and incubation for 24 h.

The silver nanoparticles (AgNPs) synthesized using *Trametes versicolor* were tested for antibacterial activity against selected Gram-positive bacterial strains (*Staphylococcus aureus* and *Staphylococcus epidermidis*) and Gram-negative strains (*Escherichia coli* and *Pseudomonas aeruginosa*). We tested two types of nanoparticles: AgNPs 1 (nanoparticles obtained in static culture) and AgNPs 2 (nanoparticles obtained in shaking culture). The antibacterial potential of silver nanoparticles is shown in Figure 2 and Figure 3.

The experimental results showed that AgNPs 2 has a stronger effect on the tested bacterial strains. Higher activity of the tested compounds against Gram-negative strains was also observed. In the case of Gram-positive bacteria, it was necessary to use higher concentrations of compounds. The use of concentrations of both tested nanoparticles below 5 µg/mL reduced the growth of *S. aureus* from 20 to 60%. Moreover, their slightly lower activity against *S*. *epidermidis* was noted. At higher concentrations, the tested silver nanoparticles caused 80–90% growth inhibition of the Gram-positive microorganisms. *E. coli* was the most sensitive strain to the tested compounds. The bactericidal effect was obtained at a concentration of 20 µg/mL for AgNPs 1 and 7 µg/mL for AgNPs 2. At lower concentrations of both compounds (4–5 µg/mL), satisfactory results were also obtained for *E. coli* (50–60% growth inhibition). Nanoparticles also showed good antibacterial activity against *P. aeruginosa*. However, the strain was less sensitive compared to *E. coli*. When higher concentrations of nanoparticles (above 10 µg/mL) were added to the *P. aeruginosa* culture, the growth inhibition was 80%.

Due to the increasing number of multidrug-resistant organisms, the search for alternatives to antibiotics has been a recent theme of research. Silver nanoparticles are considered alternative sources to overcome the threat of antibacterial resistance [42]. We have shown that silver nanoparticles (AgNPs) produced using *T. versicolor* have stronger activity against Gram-negative bacteria. The lower sensitivity of Gram-positive bacteria to AgNPs may be because the peptidoglycans, which are components of the cell wall, act as a barrier preventing the internalization of the nanoparticles [43]. A similar effect was obtained with AgNPs synthesized using *Gloeophyllum striatum*, which were active against *E. coli* and *P. aeruginosa* [44]. Singh et al. (2013) described the extracellular synthesis of AgNPs using *Penicillium* spp. In this case, the nanoparticles showed antimicrobial activity against *E. coli* and *S. aureus* strains [45]. Ahluwalia et al. (2014) synthesized silver nanoparticles using *T. harizanum*, which showed great antimicrobial potential against *S. aureus* and *K. pmeumoniae* [46]. Silver nanoparticles produced using white rot fungi *Schizophyllum radiatum* showed antibacterial activity against *E. coli*, *K. pneumoniae*, *E. aerogenes*, *P. aeruginosa*, *S. aureus*, and *S. paratyphi* [47]. Other research described spherical silver nanoparticles synthesized using *A. tubingiensis* and *B. ochraleuca*. Nanoparticles produced using *A. tubingiensis* were more effective compared to the other fungus, inhibiting 98% of *P. aeruginosa* growth at 0.28 µg/mL [41]. Halkai et al. (2018) showed that AgNPs synthesized using *Fusarium semitectum* emerged as novel antimicrobial agents against the most resistant endodontic pathogens, *E. faecalis* [48]. Other studies described silver nanoparticle-treated cotton as having high antimicrobial activity against *S. aureus* and *E. coli*. The AgNPs were produced using *Alternaria Alternata* [49]. It has also been proven that AgNPs can exert effects on tumor cells. Nanoparticles synthesized using *Fusarium oxysporum* exhibited antibacterial (against *E*. *coli* and *S. aureus*) and antitumor (human breast adenocarcinoma) potential [50]. 

### 2.3. Combined Antimicrobial Activity of AgNPs and Phytocompound

Taking into account the problem of the risk of cytotoxicity of silver nanoparticles (AgNPs), combined therapy was employed using a phytochemical, which was aimed at reducing the doses of nanoparticles.

**Figure 4:**
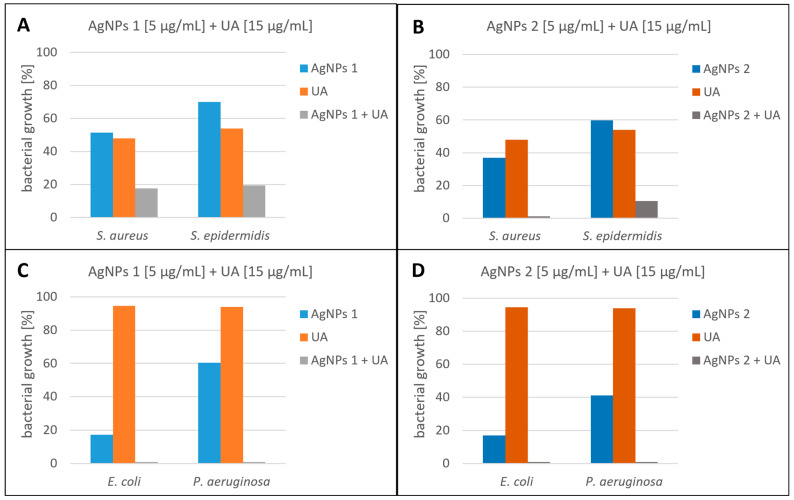
Synergistic effect of AgNPs and ursolic acid against Gram-positive bacteria: *Staphylococcus aureus* and *Staphylococcus epidermidis* ((**A**) AgNPs1 and (**B**) AgNPs 2) and Gram-negative bacteria: *Escherichia coli* and *Pseudomonas aeruginosa* ((**C**) AgNPs 1 and (**D**) AgNPs 2).

Based on our previous research, we chose ursolic acid (UA), which showed an antimicrobial potential [51]. We tested the antimicrobial synergy of AgNPs and UA. The results are presented in Figure 4. The most representative synergistic effect was observed with the treatment of 5 µg/mL nanoparticles (AgNPs 1 and AgNPs 2) in combination with 15 µg/mL UA against *E. coli* and *P. aeruginosa* with a bactericidal effect (Figure 4C,D). Moreover, satisfactory results were obtained for Gram-positive strains, where the addition of UA significantly enhanced the effect of nanoparticles. The coadministration of AgNPs considerably reduced the growth of both *Staphylococcus* strains, with a bactericidal effect against *S. aureus* (AgNPs 2 + UA). The weakest antibacterial activity of the tested compounds was observed for *S. epidermidis*. When AgNPs and UA were tested individually, the growth of *S. epidermidis* was inhibited only by 30–40% and 46%, respectively.

Synergistic approaches combine two or more compounds to result in higher efficiency compared to that of any individual substances [52]. In our work, the synergistic effects of biogenic silver nanoparticles in combination with a phytocompound are described for the first time. Literature data have reported the simultaneous action of silver nanoparticles (AgNPs) and antibiotics. However, our goal was to use a safe and natural compound (such as ursolic acid) to reduce the concentrations of nanoparticles used. We obtained very satisfactory results. Fayaz et al. (2010) studied the synergistic antimicrobial activity of AgNPs synthesized using *Trichoderma viride* against some Gram-positive and Gram-negative bacteria. The results showed that the antibacterial potential of ampicillin, kanamycin, erythromycin, and chloramphenicol increased in the presence of nanoparticles against tested bacteria [53]. In other studies, the synergistic effects of antibiotics (azithromycin, cefuroxime, cefotaxime, and fosfomycin) against *E. coli* and *Salmonella* spp. were increased in the presence of AgNPs (synthesized in the aqueous extract of *Ulva fasciata* alga) compared to antibiotic only [54]. They also observed an antagonistic effect. The antimicrobial activity of nanoparticles with oxacillin and neomycin against *S. aureus* was significantly decreased compared to antibiotics only [54]. Other studies reported the combination of AgNPs (achieved using the propolis extract) with bacteriophage as an antimicrobial agent. This combination offers nanoparticles with enhanced antimicrobial potential [55].

### 2.4. Bacterial Cell Viability Assays

The viability of bacterial cells incubated with silver nanoparticles and/or ursolic acid was studied using the Alamar Blue reagent. The test involved measuring fluorescence resulting from the conversion of non-fluorescent resazurin to highly fluorescent resorufin. Resazurin, the active ingredient in Alamar Blue, can only be reduced to resorufin by living microorganisms [56]. The study aimed to assess the impact of the tested compounds and their combinations on bacteria survival.

**Figure 5:**
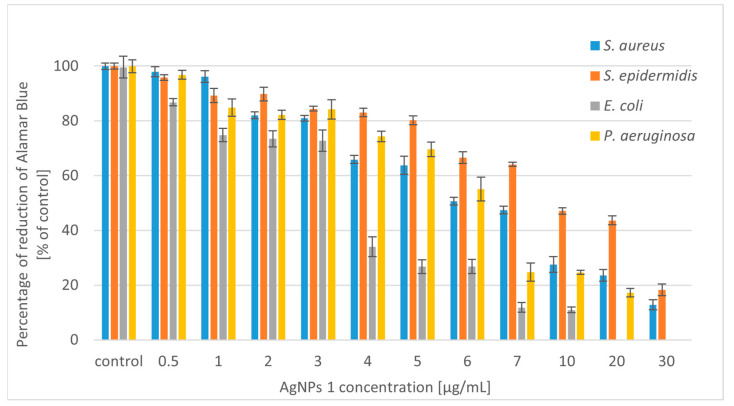
Percentage reduction of Alamar Blue by bacteria incubated with AgNPs 1.

**Figure 6:**
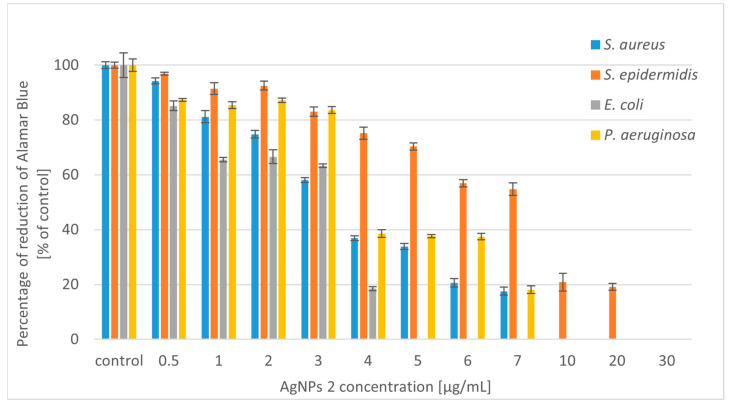
Percentage reduction of Alamar Blue by bacteria incubated with AgNPs 2.

**Figure 7:**
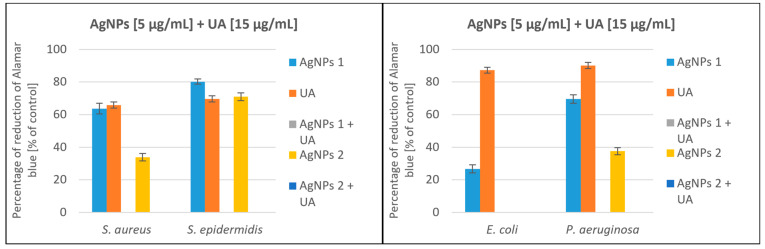
Percentage reduction of Alamar Blue by bacteria incubated with AgNPs 1 or AgNPs 2 and ursolic acid.

A significant decrease in the viability of tested bacteria was noted after incubation with a high concentration of silver nanoparticles (AgNPs) (Figure 5 and Figure 6). Simultaneous applications of AgNPs and ursolic acid (UA) decreased cell viability to zero in the culture of all tested strains (Figure 7). We observed a correlation between the inhibition of Alamar Blue reduction and bacterial growth inhibition. These results confirm the strong synergistic effect of both tested compounds.

## 3. Materials and Methods

### 3.1. Materials

The strain *Trametes versicolor* IM 373 originated from the Microorganisms Collection of the Department of Industrial Microbiology and Biotechnology, University of Lodz, Poland. Silver nitrate and deionized water (LC–MS Grade) LiChrosolv^®^ were obtained from Merck (Warsaw, Poland). Sabouraud dextrose broth and Mueller Hinton broth were obtained from Becton Dickinson (Warsaw, Poland). Thermo Scientific™ Nalgene™ Rapid-Flow™ Sterile Single Use Vacuum Filter Units were acquired from Thermo Scientific™ (Warsaw, Poland). DMSO was obtained from BioShop (Burlington, ON, Canada). Silicon wafers were obtained from Agar Scientific (Stansted, UK). Alamar Blue Cell Viability Reagent supplied by ThermoFisher Scientific (Warsaw, Poland).

### 3.2. Methods

#### 3.2.1. Synthesis of Silver Nanoparticles Using *Trametes versicolor* IM 373

The *Trametes versicolor* strain was inoculated on agar plates and incubated for 7 days at 28 °C. After incubation, the agar with mycelium was cut into 12 pieces and placed in a flask containing 300 mL of Sabouraud dextrose medium. *T. versicolor* was incubated for 7 days at 28 °C in the dark (one culture in static conditions and the other on a rotary shaker at 140 rpm). Then, the mycelium was filtered through sterile filter paper, and the post-culture liquid was transferred to a sterile flask. The *T. versicolor* filtrate was supplemented with a stock solution of silver nitrate. The final concentration of silver ions in the fungus filtrate was 1 mM. Controls were performed without the addition of silver nitrate. The prepared samples were incubated for 5 days at 8 °C in the dark under shaking conditions (magnetic stirrer). After incubations, the samples were centrifuged for 20 min at 9000 RPM. The obtained sediment of nanoparticles was suspended in 5 mL of deionized water and micronized in an ultrasonic cleaner for 30 min. Then, the samples were centrifuged, and the sediment was dried. Characterization of obtained nanoparticles was carried out using UV–VIS spectroscopy and scanning electron microscopy (SEM).

The process of silver ion reduction was observed on the basis of changes in the UV–“VIS spectrum to achieve the peak maximum in the area characteristic for nanoparticles. Monitoring of silver ion reduction was carried out via UV-VIS spectroscopy using a UV–VIS spectrophotometer Specord 200 (Analytik Jena, Jena, Germany) operating in absorbance mode at wavelengths from 200 to 1000 nm at a resolution of 2 nm.

The silver nanoparticles were estimated via scanning electron microscopy (SEM) using a Nova NanoSEM scanning electron microscope (FEI, Hillsbro, OR, USA). The silver nanoparticles were spread on a silicon wafer and dried at room temperature in the dark. The images were achieved in immersion mode using a circular backscatter detector (CBS) at 5.00 kV acceleration at a magnification of 100,000×.

#### 3.2.2. Evaluation of the Antibacterial Activity of Silver Nanoparticles

The antimicrobial activity of silver nanoparticles (AgNPs) was evaluated using the microdilution method for aerobic bacterial strains according to the CLSI documents M07 (11th Edition) [57]. The antimicrobial activity of the nanoparticles was determined against aerobic bacteria (*Staphylococcus aureus* ATCC 6538, *Staphylococcus epidermidis* ATCC 12228, *Escherichia coli* ATCC 25922, and *Pseudomonas aeruginosa* ATCC 27853). The growth of the aerobic bacterial strains that were either treated with the silver nanoparticles or left untreated was evaluated in 96-well microtiter plates in Mueller–Hinton broth. The silver nanoparticles were supplemented in a range of 1–30 µg/mL, and their antimicrobial potential was determined. They were diluted in the appropriate growth medium before administration. An inoculum of bacteria grown in the Mueller–Hinton broth was added to each well to achieve a final density of 5 × 10^5^ CFU/mL. The microtiter plates were then incubated for 24 h at 37 °C. After incubation, the optical density was measured spectrophotometrically at 620 nm. Experiments were performed in triplicate, leading to the analysis of six independent experiments. The antimicrobial activity of the AgNPs was calculated as the percentage of bacterial growth inhibition (SD) compared to that of the biotic control (bacteria incubated in the medium).

The combined antibacterial activity of silver nanoparticles (AgNPs 1 or AgNPs 2) and ursolic acid (UA) was investigated using the same method.

#### 3.2.3. Bacterial Cells Viability Assays

Analyses of bacterial cell viability after 24 h of incubation with silver nanoparticles (AgNPs) or AgNPs/ursolic acid were performed using Alamar Blue reagent [56]. Control cultures and cultures with the addition of the tested compounds were prepared as described in Section 3.2.2. Alamar Blue^®^ (volume—5 µL) was added to each well with 24-h bacterial cultures and then incubated for 40 min at 37 °C. The reduction in Alamar Blue^®^ is shown as a percentage of the control group (±SD) after fluorescence measurements at λ = 540 nm using a multimode microplate reader BMG 139 LabTech FLUOstar Omega (BMG LABTECH GmbH, Ortenberg, Germany).

## 4. Conclusions

The biological synthesis of silver nanoparticles (AgNPs) offers a sustainable and eco-friendly approach to nanoparticle production. The use of biological agents as reducing and stabilizing agents provides several advantages, including eco-friendliness, cost-effectiveness, versatility, and biocompatibility. Silver nanoparticles synthesized using biological methods have shown great potential in biomedical applications, environmental remediation, and catalysis. In the presented study, the white rot fungus *T. versicolor* was used for the production of AgNPs. Such a synthesis type may be considered eco-friendly as it is free from any toxic chemicals or organic solvents during the biosynthesis process. The high antimicrobial potential of the tested AgNPs was confirmed. Combined therapy using ursolic acid was used for the first time, which allows for the reduction of the doses of nanoparticles. With further research and development, the biological synthesis of AgNPs is expected to play a significant role in advancing nanotechnology and its diverse applications. Moreover, it is very important to explore new approaches to eliminate pathogens, including reducing the amount of antibiotics used, to overcome the current emergency of drug resistance. Filamentous fungi are promising candidates for silver nanoparticle production because of the simplicity and low cost of their culture.

## Data Availability

The data presented in this study are available on request from the corresponding author.
